# Trends and disparities in self-reported past-month poor mental health among individuals using cannabis in the U.S., 2016–2023

**DOI:** 10.1007/s00127-026-03145-w

**Published:** 2026-06-11

**Authors:** Xiangxiang Jiang, Gang Lv, Minghui Li, Jing Yuan, Z. Kevin Lu

**Affiliations:** 1 Department of Clinical Pharmacy and Outcomes Sciences, College of Pharmacy, University of South Carolina, 715 Sumter Street, Columbia, SC USA; 2 Department of General Surgery, The First Medical Center of Chinese PLA General Hospital, Beijing, China; 3 Department of Clinical Pharmacy and Translational Science, University of Tennessee Health Science Center, Memphis, TN USA; 4 Institute of Chinese Medical Sciences, University of Macau, N22 Avenida da Universidade, Taipa, Macau China

**Keywords:** Cannabis Use, Mental Health, Trends, Disparities, Behavioral Risk Factor Surveillance System (BRFSS)

## Abstract

Using 2016–2023 data from the Behavioral Risk Factor Surveillance System (BRFSS), this study examined trends in self-reported past-month poor mental health (≥ 1 day) among individuals using cannabis and disparities in subgroups. From 2016 to 2023, the prevalence of self-reported past-month poor mental health (≥ 1 day) among those using cannabis increased from 54.54% to 67.88%. Women reported higher rates than men (77.08% vs. 60.14%). Young adults (25–34) showed the largest increase (from 51.42% to 77.67%). Hispanics (69.17%) exhibited the highest prevalence in 2023. Findings underscore the need for mental health screening and targeted counseling as cannabis legalization expands.

**Supplementary Information:**

The online version contains supplementary material available at https://doi.org/10.1007/s00127-026-03145-w.

## Introduction

Cannabis is the most commonly used federally illicit substance in the U.S., [1] and its use has increased rapidly over the past decade. Prior research has shown that cannabis use is associated with mental health issues, including depression, anxiety, and psychosis. [2] However, population-level data examining recent mental health trends among individuals using cannabis are limited. Moreover, disparities in cannabis-related mental health outcomes remain poorly understood.

As more states legalize medical and recreational cannabis, these shifts have increased the risks of cannabis use disorders (CUD), raising concern over the potential adverse mental health consequences. Understanding these trends is critical for prevention and public health policy as legalization continues to expand.

This study aimed to (1) evaluate national trends in the prevalence of self-reported past-month poor mental health (≥ 1 day) among individuals using cannabis from 2016 to 2023, and (2) identify disparities by mode of consumption, age, sex, race and ethnicity. Past-month poor mental health (≥ 1 day) was defined as a self-report of at least one day in the past 30 days when mental health was “not good”, including stress, depression, and problems with emotions.

## Methods

### Data source and study population

We analyzed data from the 2016–2023 Behavioral Risk Factor Surveillance System (BRFSS). [3] BRFSS is a large, state-based, nationally representative telephone survey that collects data on health-related risk behaviors, chronic conditions, and use of preventive services among U.S. adults aged ≥ 18 years. Conducted annually across all 50 states, the District of Columbia, and U.S. territories, it completes over 400,000 interviews each year using random-digit dialing of both landlines and cell phones.

This study included 865,178 unweighted respondents, corresponding to a weighted population of 448,137,324 respondents.

### Measurements

Cannabis use was measured by the survey question: “During the past 30 days, on how many days did you use marijuana or cannabis?”. Responses were recorded as: (1) Number of days (1–30); (2) None; (3) Don’t know/Not sure; (4) Refused; (5) Not asked or Missing. Respondents who reported using cannabis on at least one day in the past 30 days were classified as individuals using cannabis. Those who answered “None” were categorized as non-users. Frequent cannabis use was defined as use on more than 6 days in the past 30 days, and daily cannabis use was defined as use on all 30 days. Individuals using cannabis were further classified by consumption mode: smoking, eating, vaping, dabbing, or other. The exact wording of these items and response options is provided in Supplementary Table 2.

Past-month poor mental health was assessed by the question: “Now thinking about your mental health, which includes stress, depression, and problems with emotions, for how many days during the past 30 days was your mental health not good?”. Responses were recorded as: (1) 1–30 days; (2) None; (3) Don’t know/Not sure; (4) Refused; (5) Not asked or Missing. Respondents who reported one or more days of poor mental health were classified as having past-month poor mental health (≥ 1 day).

Participants with responses of “Don’t know/Not sure”, “Refused”, or missing were excluded from the analysis.

### Data analysis

This study used BRFSS sampling weights, generated by raking on age, sex, race and ethnicity, education, marital status, telephone source, and home ownership, to produce nationally representative estimates. [3] Analyses were stratified by mode of use, age, sex, race and ethnicity. A weighted chi-square test was used to compare the prevalence in 2016 and 2023.

To evaluate the association between different cannabis use patterns and self-reported past-month poor mental health (≥ 1 day), weighted multivariable logistic regression was used, adjusting for study year, age, sex, race and ethnicity, education, marital status, income, and employment.

## Results

A total of 865,178 unweighted respondents were included, representing 448,137,324 weighted respondents; 11.33% reported past-month cannabis use. (Supplementary Table 1) Compared to those not using cannabis, individuals using cannabis were more likely to report past-month poor mental health (≥ 1 day) (61.34% vs. 35.14%, *P* < 0.001).

**Fig. 1:**
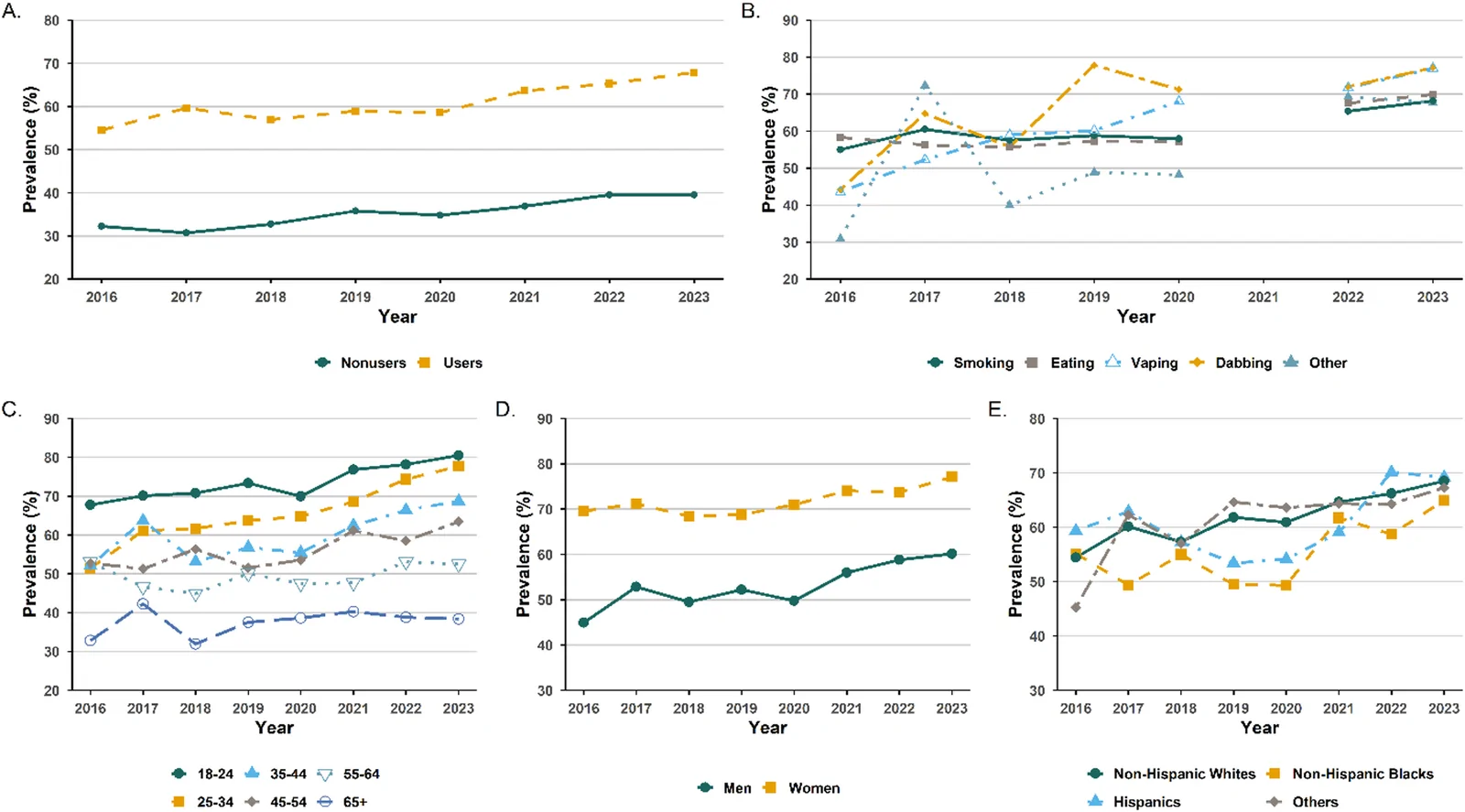
Trends in weighted prevalence of self-reported past-month poor mental health (≥ 1 day) among U.S. adults, 2016–2023. **A**. Trends by cannabis use status; **B**. Trends by consumption mode; **C**. Trends by age group; **D**. Trends by sex; **D**. Trends by race and ethnicity

Individuals using cannabis reported consistently higher prevalence of past-month poor mental health (≥ 1 day) than those not, increasing from 54.54% in 2016 to 67.88% in 2023 (*P* < 0.001). (Fig. 1A & Supplementary Table 2) Prevalence rose across all modes of use (*P* < 0.05), with higher increases among vaping (76.96% in 2023) and dabbing (77.18% in 2023). (Fig. 1B)

Cannabis use was associated with 131% higher odds of self-reported past-month poor mental health (≥ 1 day) (OR: 2.31; 95% CI: 2.23–2.39). (Supplementary Table 3) Frequent cannabis use (≥ 6 days) was also associated with increased odds (OR: 1.21; 95% CI: 1.13–1.29). However, daily cannabis use was not significantly associated with self-reported past-month poor mental health (≥ 1 day) (OR: 0.95; 95% CI: 0.89–1.02).

The prevalence of self-reported past-month poor mental health (≥ 1 day) increased rapidly across all age groups aged 18–54 (*P* < 0.05). (Fig. 1C) Alarmingly, among those aged 18–24, the prevalence rose from 67.74% to 80.49% (*P* = 0.001); while rates among those aged 25–34 experienced the largest rise, from 51.42% to 77.67% (*P* < 0.001), a 51% increase over just 7 years.

Women consistently had a higher prevalence of self-reported past-month poor mental health (≥ 1 day) compared with men, but the difference was decreasing. (Fig. 1D) From 2016 to 2023, prevalence among women increased from 69.54% to 77.08% (*P* < 0.001), while among men it rose from 44.95% to 60.14% (*P* < 0.001).

Prevalence increased across all racial and ethnic groups (*P* < 0.001). (Fig. 1E) In 2023, Hispanics reported the highest prevalence (69.17%), followed by non-Hispanic Whites (68.48%). From 2016 to 2023, the greatest relative increase occurred among other races (45.26% in 2016 to 67.25% in 2023, a 48.60% increase rate), while non-Hispanic Blacks showed the slowest growth (55.13% in 2016 to 64.96% in 2023, a 16.55% increase rate) and the lowest overall prevalence (64.96% in 2023).

## Discussion

From 2016 to 2023, individuals using cannabis experienced a rising and disproportionate burden of self-reported past-month poor mental health (≥ 1 day), with the highest prevalence among those using high-potency modalities, young adults, women, and Hispanics. Use of vaping and dabbing–modes associated with higher Δ9-tetrahydrocannabinol (THC) exposure, was linked to greater prevalence, underscoring potential risks of high-potency products. [4] Additionally, past-month cannabis use and frequent use (≥ 6 days) were associated with higher odds of past-month poor mental health (≥ 1 day), whereas daily use (30 days) was not associated.

Younger adults (18–24) showed the highest prevalence of self-reported past-month poor mental health (≥ 1 day), which is consistent with prior evidence from a systematic review and meta-analysis indicating that cannabis use in young populations is associated with increased risks of depression, anxiety, and suicidal behaviors, [5] potentially reflecting neurodevelopmental vulnerability of the brain during young adulthood. [6] Poorer outcomes among women may reflect hormonal influences, metabolic differences, and endocannabinoid variation. [7] Notably, women generally report higher prevalence of mental health problems irrespective of cannabis use status and may be more likely to report mental health symptoms in survey-based data, which could partly explain the observed differences. Hispanic users had the highest burden, likely reflecting both baseline disparities in mental health [8] and structural barriers to care, including unfair treatment and limited awareness of services. [9].

Adults aged 25–34 showed the largest relative increase, possibly reflecting persistent cannabis-related vulnerability across young adulthood, including cannabis use to cope with negative emotions, which has been linked to poorer mental health. [10] Other racial groups, including Asian, Native American, Pacific Islander, and multiracial individuals, experienced the steepest increases, probably reflecting expanded cannabis availability after legalization due to higher cannabis retail density [11] and persistent barriers to culturally competent care. [9].

This study has several limitations. First, the cross-sectional design limits causal inference; it cannot determine whether cannabis use preceded reporting past-month poor mental health (≥ 1 day). Second, BRFSS relies on self-reported data, which may be subject to recall bias and social desirability effects, potentially underestimating true cannabis use or mental distress. Third, there are measurement limitations. The survey does not measure cannabis potency, dosage, or type of product, limiting assessment of potency-related effects. The survey uses a single-item measure of past-month poor mental health, which may not capture its multidimensional nature. Finally, because state-level policy data were not linked at the respondent level, variations by legalization environment should be interpreted cautiously.

Clinicians should incorporate screening for cannabis use and related mental health symptoms into routine care, especially for younger adults, women, and individuals using high-potency products. The sharpest increases observed among adults aged 25–34, Hispanic and other racial populations, underscore the need to strengthen early adult mental health programs and improve access to culturally competent care.

## Conclusions

Prevalence of self-reported poor mental health (≥ 1 day) among individuals using cannabis has increased substantially from 2016 to 2023, with disproportionate impacts on young adults, women, and racial and ethnic minorities. This nationally representative study adds timely evidence by characterizing recent trends and disparities across sociodemographic groups and cannabis use modes. As legalization expands, coordinated clinical screening, public education, and regulatory actions are urgently needed to address the mental health risks associated with frequent and high-potency cannabis use.

## Supplementary Information

Below is the link to the electronic supplementary material.

Supplementary Material 1

## Data Availability

The data that support the findings of this study are publicly available from the U.S. Centers for Disease Control and Prevention (CDC) through the Behavioral Risk Factor Surveillance System (BRFSS). No additional unpublished data are available from the authors.
